# Body lice: a vector for re-emerging disease outbreak in a rehabilitation camp in Northwestern Iran

**DOI:** 10.1186/s13104-024-06709-8

**Published:** 2024-02-20

**Authors:** Esmail Ghorbani, Eslam Moradi-Asl, Mustapha Ahmed Yusuf

**Affiliations:** 1 Arthropod-Borne Diseases Research Centre, Ardabil University of Medical Sciences, Ardabil, Iran; 2 Department of Medical Entomology and Vector Control, School of Public Health, Tehran University of Medical Sciences, Tehran, Iran; 3 Department of Clinical Microbiology, Aminu Kano Teaching Hospital, Kano, Nigeria

**Keywords:** Body louse, Outbreak, Re-emerging, Northwestern Iran, Rehabilitation camp

## Abstract

**Objective:**

The report of the outbreak of body louse in northwestern Iran after three decades reminds us again of the danger of the re-emerging of previous epidemics.

**Results:**

The results of the study that nearly 70% of the patients in a rehabilitation Centre were infected with body louse. In this study, scientific measures were taken to prevent the spread of body lice to healthy people, including isolation of the patients, washing the clothes of those infected at high temperatures, and spraying the rest area, beddings, and blankets. This is a more recent report on an outbreak of body louse in Iran in 2023.

## Introduction

Human pediculosis disease is defined as the presence of eggs, nymphs, or adults lice on the body or head which is known more than 10,000 years ago. Human pediculosis is a public health problem worldwide [1]. About 550 species of blood-sucking lice have been described, these lice are currently assigned to 50 genera and 15 families [2]. The transmission of lice occurs through close contact such as head-to-head, changing hats or scarves and shawls, and changing clothes or pillowcases [3]. Among the three common genera of lice, body louse is the vector of important epidemics such as epidemic typhus, epidemic relapsing fever, and trench fever in the world, which has been associated with high morbidity and mortality (4–5). According to one review, body lice are prevalent in homeless populations, refugees, migrants, and war-affected populations in many countries, including France, Spain, Italy, Ethiopia, Kenya, Uganda, and Colombia [2]. Body lice can also be found in school children, travelers, and military personnel [3]. In Iran, body lice infestations have been reported in different regions and populations, such as rural areas, nomadic tribes, prisoners, and war veterans. A study conducted in 2018 found that 11.4% of 1050 rural residents in Kermanshah Province were infested with body lice [2, 4]. Another study conducted in 2019 found that 6.8% of 103 nomadic tribes in Fars Province were infested with body lice. Body lice have also been detected in prisoners in Tehran and war veterans in Ahvaz [2, 4].

## Materials and methods

**Fig. 1:**
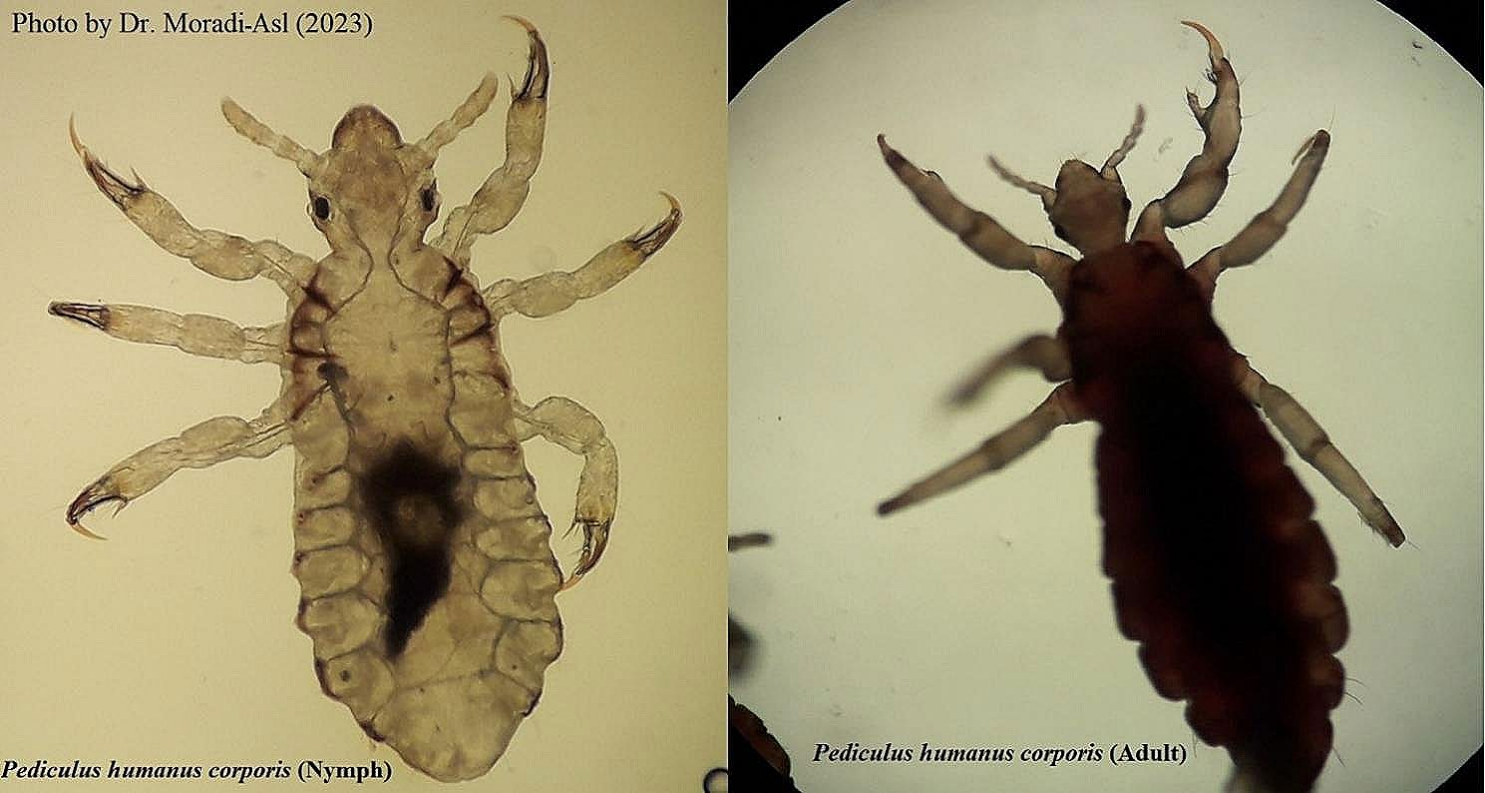
Morphological Characteristics of body louse nymphs and adults in a Rehabilitation Camp in Northwestern Iran

**Fig. 2:**
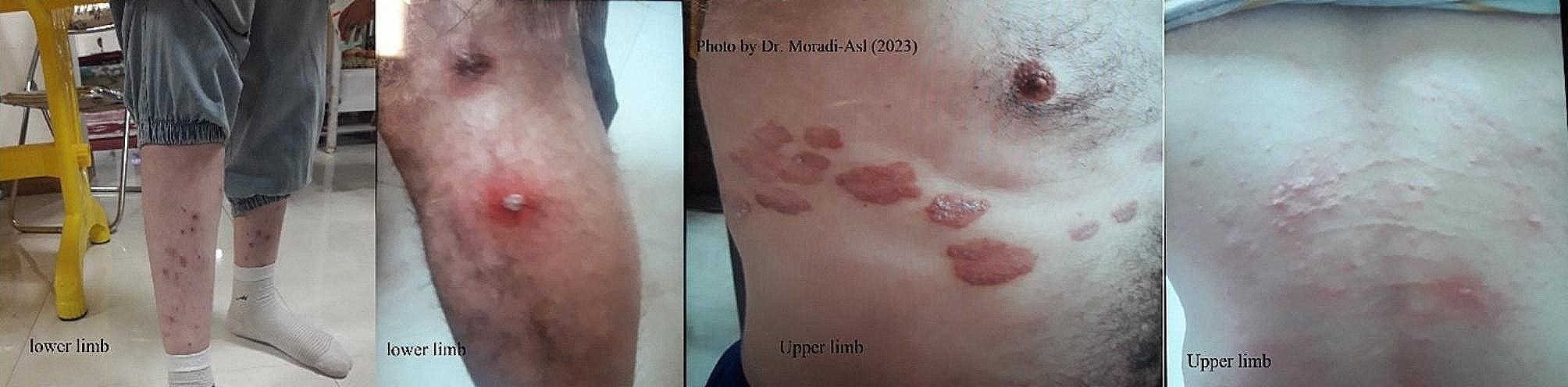
Different sites of the louse bite on the body in a Rehabilitation Camp in Northwestern Iran

The morphological characteristics identified include short and thick leg, short thumb-like spine at the end of the inner side as well as a short claw and a large curved claw [6] (Fig. 1). Following an outbreak of body louse in a rehabilitation home in Ardabil City in November 2022, all the necessary medical entomological investigations were carried out to establish details of the outbreak. This camp has 250 male patients who were divided into two sections, youth and middle-aged. The clothes and bodies were examined by medical entomology experts and the main location of wounds was observed in the body and legs of the patients (Fig. 2).

## Results and discussion

**Fig. 3:**
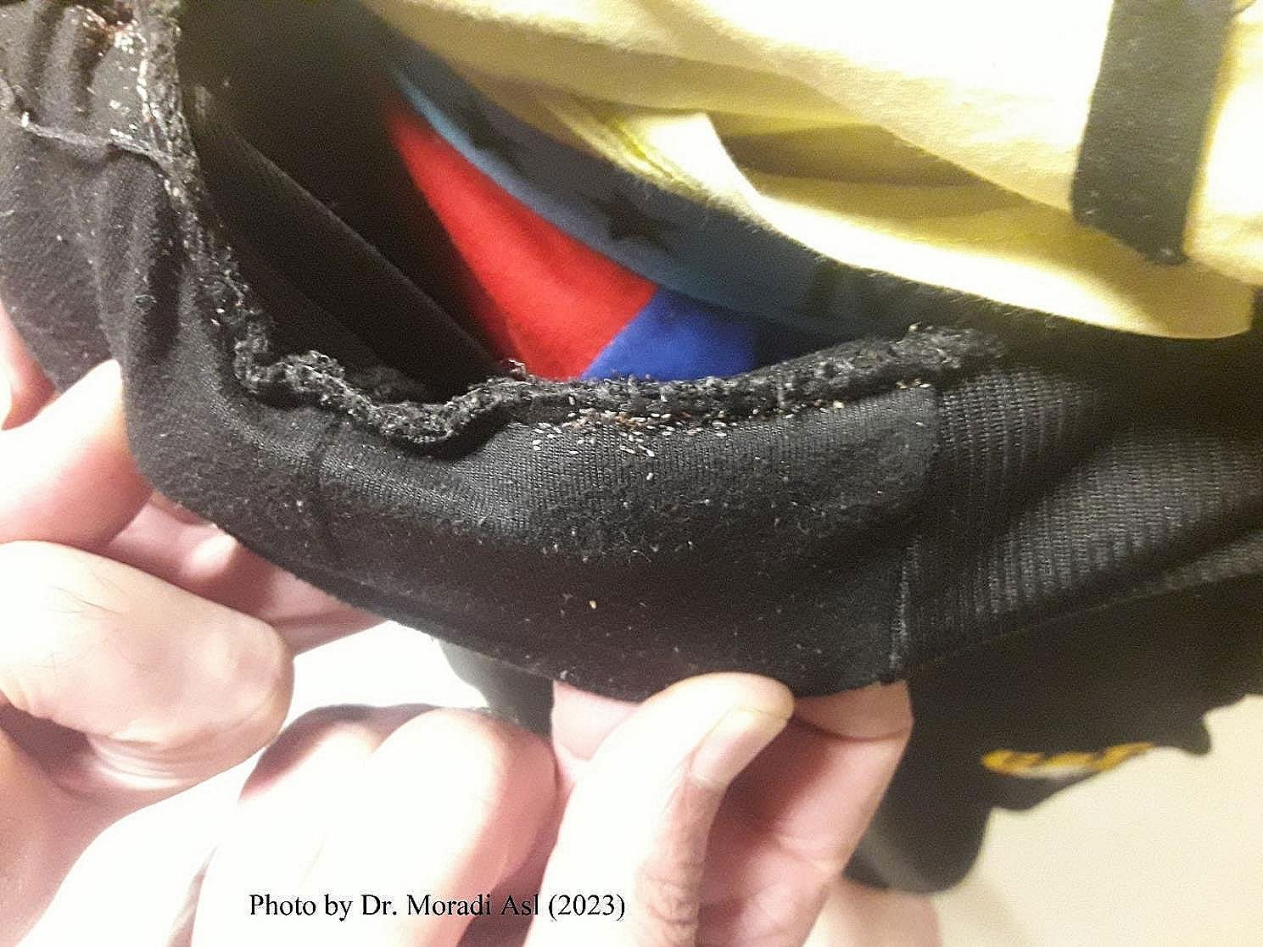
Infestation of clothes of patients with eggs in a Rehabilitation Camp in Northwestern Iran

A total of 250 patients in the camp were examined for lice infestation. One hundred and seventy-three (69.2%) contained eggs, nymphs and adults’ lice at a very high level of infestation. The lice samples were transferred to the biology and vector control laboratory of the Faculty of Health for the morphological survey. Furthermore, the location of the louse in the clothes was confirmed (Fig. 3). In terms of the state of infestation in various body parts, 60% was in the upper body and 40% in the lower body, which caused sores round the bites area due to extensive itching (Fig. 2). Body lice infestation is associated with poor social and economic status [7]. The results of the investigation of the camp condition showed overcrowding, lack of timely changing and washing of clothes, blankets and sheets. There was no periodic environmental health visit by the ministry of health. The lack of access to detergents and lack of personal hygiene, including personal towels, hair removal, and bathing, are the most important factors in the re-emerging of the body lice. The control measures taken for this camp include training staff and clients, washing all clothes at high temperatures, and spraying contaminated clothes, sheets, and beds with Deltamethrin 5% [8].

### Limitation of the study

This study was a report of an outbreak of body louse in a rehabilitation Centre, although, it involves a sizeable number of clients, however, it may not be enough to make a generalization into the wider population. We ought to have included more centers at different regions when this outbreak was ongoing, due to financial constraints we couldn’t do that.

## Data Availability

Data is available upon request with the corresponding author.
